# Health research systems in Somaliland: a qualitative study on perspectives of government and non-governmental staff

**DOI:** 10.3389/frhs.2023.1225141

**Published:** 2023-11-23

**Authors:** Soheir H. Ahmed, Jonah Kiruja, Ayanle Solieman, Cynthia Khamala Wangamati

**Affiliations:** ^1^ College of Medicine and Health Science, University of Hargeisa, Hargeisa, Somaliland; ^2^ Department of Community Medicine and Global Health & Center for Medical Ethics, University of Oslo, Oslo, Norway

**Keywords:** health research systems, governmental organisations, non-governmental organisations, stakeholders, Somaliland

## Abstract

**Background:**

Globally, the importance of effective national health research systems has gained considerable attention. Literature indicates low research output in Africa; Africa accounts for only 2% of the world's research output and 1.3% of global publications. In Somaliland, where provision of quality healthcare services is crucial, understanding and enhancing the health research system is a critical endeavor.

**Aim:**

The aim of this study is to explore the perspectives of government and non-governmental stakeholders on the health research systems in Somaliland.

**Method:**

The study employed an exploratory qualitative study design that entailed in-depth interviews with participants. Thirty-four study participants were interviewed; they included key persons in the academic and health sector, government and international and local non-governmental organisations (NGOs) involved in health research systems. A semi structured interview guide was used to conduct the in-depth interviews with purposively selected participants. The collected data was analyzed thematically.

**Findings:**

We found that there was no national health research center in Somaliland. The country also lacked a national health research policy. There was limited funding for research, funds were mostly from international organisations and researchers' own funds. In addition, staff working in research centers were ill equipped to conduct research and study participants highlighted the need for national health research governance.

**Conclusion:**

This study highlights the importance of health research systems in Somaliland. We recommend the establishment of a national health research institute, development of a national health research policy and priorities, allocation of sufficient and sustainable funding, capacity building of staff and strengthening of the national health research governance in health research systems.

## 1. Introduction

Global and regional tools such as the World Health Assembly, Bamako Call for Action, the 1998 forty-eighth World Health Organization (WHO) Regional Committee for Africa, and the 2008 Algiers Declaration encourage countries to strengthen national health research systems (1). Contextualized healthcare interventions are vital to reduction of morbidity and mortality, hence the need for strengthening national health research systems to respond to local health challenges (2, 3). The WHO describes the national health research systems (NHRS) as a structure that provides governance, capacity building, knowledge generation and platform to use evidence, in an environment with sustainable funding mechanisms (4). National health research system functions include but are not limited to governance; developing and sustaining research capacities; producing and using research knowledge; and resource mobilization for and financing of research (5).

Literature indicates low research output in Africa; Africa accounts for only 2% of the world's research output (4, 6) and 1.3% of global publications (5, 7). The limited research capacity in African countries can be attributed to misalignment of research funding with national priorities, lack of optimal coordination among stakeholders, limited health research capacity (7, 8), inadequate funding, skills, infrastructure, and weak health research governance (9).

A study of the state of health research governance in Africa indicated that 18 out of the 35 Member States of the WHO African Region had legislation to regulate the conduct of health research, 12 countries lacked legislation and for the remainder, legislation was either grossly outdated or too limiting in scope, while some countries had multiple laws (10). The study reported that out of the 35 countries, health research policies and strategies were in place in 16 and 15 countries, respectively, while research priority lists were available in 25 countries. Overlapping mandates of institutions responsible for health research partly explained the lack of strategic documents in some countries. Twenty-five of the 35 countries had a focal point and unit within the Ministries of Health (MoH) to coordinate research (10).

To address weak health research systems in Africa, a strategy endorsed by the WHO Regional Committee for Africa was developed titled “Research for Health: a strategy for The African Region, 2016–2025” (11). The aim of the strategy is to improve national health research systems through interventions backed by recent developments in research and includes an enabling environment, sustainable financing, human resources capacity-building, knowledge translation, and effective coordination and management.

In Somaliland, the health sector strategic plan II for the years 2017–2021, and the national health policy II stress the importance of using research to better understand the health challenges in the country. The knowledge on health challenges helps in prioritizing resource allocation and capacity building within the six building blocks of the healthcare system namely: service delivery, medical drugs and technology, health financing, health management information system, competent healthcare workforce, and leadership and governance (12).

Somaliland is a low-income country with a burden of communicable and non-communicable diseases, maternal, neonatal and nutritional conditions (13). The health system in Somaliland is faced with numerous challenges including under-investment in health, poor technical capacity for research design, data collection and analysis, dissemination and uptake and use of evidence-based research (14). Consequently, the Ministry of Health decision makers and health partners lack adequate and accurate information to determine the country's priorities. In addition, there is limited high-quality evidence to produce policy briefs and evidence that can guide development of pragmatic innovative approaches to respond to health challenges. A policy framework on national health research system developed through a consultative process with key stakeholders in health is vital for advancements in healthcare and improved public health outcomes (15).

Strengthening health research systems increases the understanding of population health needs by identification of socio-cultural barriers to health services utilization, gaps between the community and the existing health system affecting provision of health services, and health system barriers (16, 17). For instance, the National Research Foundation (NRF) in South Africa, the National Commission for Science, Technology and Innovation (NACOSTI) and the Kenya Medical Research Institute (KEMRI) in Kenya, the Nigerian Institute of Medical Research (NIMR) in Nigeria and the Rwanda Biomedical Center (RBC) in Rwanda take the lead in driving the national health research agenda in their respective countries. Through such organisations, countries can advance healthcare and improve public health outcomes (15, 18).

There is limited evidence on the best approaches to enhance health research systems in Somaliland. Despite having a research department within the Ministry of Health and Development, and different health training institutions like universities conducting research activities, there is limited capacity to carry out health research in Somaliland. Furthermore, decision making regarding health challenges is not evidence-based. There is a need to understand the status of the national health research systems in Somaliland. Therefore, the aim of this study is to explore perspectives of government and non-governmental stakeholders on the health research systems in Somaliland.

## 2. Material and methods

### 2.1. Study design

The study employed an exploratory qualitative study design that entailed in-depth interviews with participants. Using the in-depth interviews, we aimed to capture the perspectives of stakeholders in the government and non-governmental institutions and gain a deeper understanding of health research systems in Somaliland.

### 2.2. Study context

The study was conducted in Somaliland, a post conflict self-declared state. The healthcare sector serves a total population of approximately 4.5 million inhabitants (19). The Ministry of Health and Development provides healthcare services to the public through the primary health units which are closer to the community, maternal and child health centers, health centers, district hospitals, regional hospitals, and the national referral hospital in Somaliland (20). Somaliland is a low-income country, with the majority of the inhabitants having a poor socioeconomic background. Different international and local non-governmental organisations are involved with programs aimed at improving the livelihoods, education, and health status of the population. The country has both public and private higher education institutions with the mandate of impacting and advancing knowledge and skills through teaching and research.

### 2.3. Study participants and recruitment

Study participants were drawn from different sectors of academia, government, local and international NGOs and were recruited using purposive sampling. The inclusion criterion was knowledge in and experience with health research systems in Somaliland. Study participants were also drawn from the six regions of Somaliland (MarodiJeh, Sahil, Awdal, Togdheer, Sool and Sanaag) to reflect different perspectives across the country. The first author contacted the different institutions and requested the administrators to provide access to eligible individuals meeting the inclusion criteria. A total of 34 participants were interviewed.

We targeted the following institutions:

1. Six government bodies: Ministries of Health, Finance and Planning, Commission for Higher Education, National Health Profession Commission.
2. The academic sector-health and medical faculties in 7 major universities and colleges in Somaliland.
3. All regional hospitals including tertiary hospitals in Somaliland.
4. Health profession associations namely: Somaliland Medical Association, Somaliland Nursing and Midwifery Association, Somaliland Pharmaceutical Association, and Somaliland Laboratory Association.
5. Key international and local NGOs implementing health programs including those involved with health research activities.

### 2.4. Data collection

The data was collected from April to August 2020. The first author conducted semi-structured in-depth interviews; a semi-structured interview guide was used to collect data. The in-depth interviews were audio recorded with consent from research participants. The audio recorded interviews took place in private office spaces where the research participants worked. The in-depth interviews were conducted in Somali lasting approximately 50–90 min. They were later transcribed verbatim by the first and second author into English.

### 2.5. Data collection instrument

The semi structured interview guide was adapted from AlKhaldi et al. (21). The study participants were asked questions on the importance of national health research systems, governance, policy, finance, production, and use of research for health, resources for health research, NHRS challenges and innovative approaches to strengthen the gaps.

### 2.6. Data analysis

The transcribed data was imported into NVIVO 12 software. Thematic inductive data analysis was applied by identifying, analyzing, and reporting patterns within data (22). The iterative inductive data analysis entailed (i) familiarization with the data, (ii) generation of initial codes, (iii) search for themes, (iv) review of themes, and (v) defining and naming of themes (23). The transcripts and field notes were read and reread several times to gain an in-depth understanding of the data and coded using NVIVO 12 by the first and second authors. The authors then developed a table of subthemes which were reviewed and deliberated upon. Table 1 indicates our coding process.

**Table 1:** Description of the coding process.

No	Themes	Codes	Quotes to support the theme
1.	Lack of a national health research institute	• Create a national health research center	*There is a need to develop or create national research center for different purposes, including health research (In-depth interview with non-governmental organisation staff, 2)*
2.	Develop national health research policy	• There is a need for a national health research policy and priorities • No national policy for health research	*The MoHD has to create a long-term strategic plan and set priorities for national health research systems. The plan should be based on three stages: current, future and sustainable plans. All this must be set at health research system and have a clear road map to investigate deeply about the burning health research issues that are causing deaths and complications in the community…… research must be dependent on indicators’ (In-depth interview with governmental staff, 7).* *I have not heard if there is national policy but if we need to develop a national policy the whole country should take part in bringing together the experts in the policy to develop a policy that can improve research (In-depth interview with academician, 3).*
3.	Funding for national health research	• Lack of budget and financial resources	*If the government doesn’t allocate a budget, encourage the researchers, doesn’t build a team to work, doesn’t get support for dissemination, then national level of health research will not be functional* *(In-depth interview with academician, 16).*
4.	Ill equipped staff to conduct health research	• Need for capacity building	*Still we need capacity building but I think we have enough people to start something (In-depth interview with non-governmental staff, 30)*
5.	National health research governance	• Need for multidisciplinary committee to govern health research.	*We can have a committee among the governmental and non-governmental stakeholders that will help to ensure the sustainability of health research projects (In-depth interview with governmental staff, 32)*

### 2.7. Ethical considerations

The study was approved by University of Hargeisa Ethical Review Board (DRCS/29/05/2020) and the Somaliland Ministry of Health (MoHD/DG:2/899/20). All study participants were informed about the aim, purpose, and benefits of the study and that participation was voluntary. Informed written consent was obtained from study participants. All the principles of research as per Helsinki declaration were observed.

## 3. Findings

A total of 34 study participants were interviewed. Majority of the study participants were aged between 30 and 50 (91.2%) and were male 27 (79%). In regard to education, the majority had a Master's degree 30 (88%). Table 2 shows the sociodemographic characteristics of study participants.

**Table 2:** Age, gender, education & sector of study participants.

Total no (34)	Frequency (%)
Age	
30–50	31 (91.2%)
51–70	3 (8.8%)
Gender	
Male	27 (79.4)
Female	7 (20.6%)
Education	
Diploma degree	4 (11.8)
Master's degree	30 (88.2%)
Sector	
Academic	18 (53%)
Government	10 (29.4%)
Local/International NGO	6 (17.6%)

Five themes emerged from our findings: (i) lack of a national health research institute, (ii) develop national health research policy, (iii) funding for national health research, (iv) ill equipped staff to conduct health research, and (v) strengthen national health research governance.

### 3.1. Lack of a national health research institute

Study participants that took part in the interviews shared a similar view that in Somaliland, there is a lack of an organized system for conducting research at the national level. Most of the participants stated that there was no national health research center in Somaliland, when asked about its existence. They expressed the need to establish a research center at the national level that will be effective in carrying out research. Academicians stated:

*There is no national health research center in Somaliland that will collect information or conduct studies, currently the Ministry of Planning is collecting needed information and publishing it in their webpage. (In-depth interview with academician, 7)*

*Health research is developmental because it seems now, we begin to learn the research but if we made an office for the research then it will become developmental. (In-depth interview with academician 4)*

### 3.2. Develop a national health research policy

Study participants said that there was no national health research strategy while others were not aware of any national health research strategy or priorities when asked about its existence. Participants highlighted the need for a national health research policy to create a roadmap on research needs that should be prioritized. According to research participants, a research policy would provide structure on research governance and resource allocation. Non-governmental staff stated:

*There is no national health research policy. In Somaliland we have the 6 building blocks of the healthcare system and research is part of it, but there is less attention on it. (In-depth interview with non-governmental staff, 7)*

*If we get a health research policy, it will assist in the health research structure, process, committees, how to disseminate, use data, how to allocate resources … the government has to make a commitment to develop a national health research policy. (In-depth interview with non-governmental staff, 11)*

### 3.3. Funding for national health research

The study participants highlighted that health research in Somaliland has limited financial resources, and the limited funding from the international community is not sustainable or sufficient. Other study participants pointed out that nationally research funds are not allocated. Most people conducting research fund themselves or source funds from the international community. One study participant mentioned that approximately 99% of the research budget comes from international organisations. Some study participants mentioned that the limited funding available is often from health services programs and not purely from a research fund. Consequently, most of the study participants stated that there is a need to establish a national health research fund that will be allocated to different institutions including universities to conduct health research. A non-governmental staff and academician explained:

*Locally we don't allocate funds for the research and mostly research is from personal initiative or aid fund from the international community. (In-depth interview with non-governmental organisation staff, 26)*

*There is a lack of funding for health research in the country. (In-depth interview with academician, 18)*

However, it is worth mentioning that one of the study participants said that there was a plan in the future to include a budget for health research by the public policy committee. A government staff said:

*In the future budgeting plans done by the public policy committee, research will be added as an important section. (In-depth interview with governmental staff, 17)*.

### 3.4. Ill equipped staff to conduct health research

The study participants said that there are insufficient staff to conduct research due to limited resources. Moreover, there is a lack of qualified staff with the expertise and capacity to conduct quality research particularly in academia and health training institutions. Some of the academicians said:

*For the last 5 years, the human resource in university has become good in terms of quality and quantity. For example, some universities offer Masters in research. However, when we compare to our human resources needs, we have insufficient capacity to conduct research. (In-depth interview with academician, 22)*

*Research skills are very weak especially in health which sometimes there is a need for experimental research or studies. (In-depth interview with academician, 18)*

*Lack or shortage of research expertise for example may be the university teachers, 1 or 2 of them may know SPSS, epi data and analysis. (In depth interview with academician 21)*

### 3.5. Strengthen national health research governance

Study participants highlighted the need for national health research governance. The participants stated the need for a multidisciplinary committee in national health research to improve communication and collaboration between health research stakeholders. In addition, the committee will ensure relevant ministries work together to strengthen health research and ensure sustainability of health-related projects. A non-governmental staff elaborated:

*There is a need for a multidisciplinary committee to improve governance of health research … with good communication and collaboration between the different health research stakeholders. (in-depth interview with non-governmental staff, 10)*

## 4. Discussion

To our knowledge, this is the first study in Somaliland to explore the perspectives of stakeholders in academia, government, and non-governmental organisations and institutions on the national health research system in Somaliland.

Our findings showed that Somaliland lacked a national health research institute. The lack of a national health research institute contributes to low research output; the poor understanding of diseases increases a country's disease burden due to poor response mechanisms (7, 24). According to extant literature, low resource settings such as Somaliland lack the infrastructure for research; to strengthen the research capacity, the country should establish the health research systems infrastructure in terms of physical assets such as offices, laboratories, computer facilities including administrative and regulatory functions (14).

According to our findings, study participants were not aware of any existing national health research policy and recommended development of a policy on research. A previous study conducted on the state of health research governance in Africa reported research health policies in sixteen out of the thirty-five studied countries (10). The national health research policy is a formal government statement that defines the national health research vision, priorities and parameters for action to address health needs, identify resources and other needs in consultation with key stakeholders such as communities, universities and local organisations (5). The national health research policy contains a situation analysis of health needs, the performance of health research, and a long-term vision of the preferred future of research. It also contains guiding principles and values, general policy objectives, policy orientations setting out the strategic direction for research, development and innovation, the implementation framework including structures, institutions, strategic partners, communities, civil society and other actors, and their roles and relationships, and monitoring and evaluation mechanisms. This study thus shows as presented in other studies (25, 26) that the lack of a national health research policy can lead to low investment in health research since priorities are not outlined (10). Somaliland needs to develop a national health research policy and identify priorities to improve health research systems for better health indicators.

Our findings indicated that research is poorly funded by the government. A similar study found that insufficient and unsustainable funding for health research contributes to poor health outcomes (27). Insufficient funding contributes to low productivity among researchers (28) and poor quality research as researchers lack resources to conduct rigorous research (29). Furthermore, the lack of funding makes it difficult to equip researchers with research skills (30). There is a need for funding in Somaliland to produce quality research and promote research productivity and better health outcomes.

Study participants stated that staff in institutions tasked with conducting research were ill equipped (31). Similar findings have been reported elsewhere (32–34). Researchers in Somaliland should be equipped with research knowledge and skills to promote research methodological rigor, the quality of research and research productivity.

Strong governance and leadership in health research are also shown to have a positive impact on the health research systems (35, 36). On the other hand, poor governance was found to be possibly contributing to poor coordination among different actors involved in health research systems (37). In this study, poor governance, lack of leadership and lack of sustainable financial resources were found as barriers to enhancing health research systems.

## 5. Strengths and limitations

A strength of this study is that perspectives were elicited from various stakeholders involved in health research in the academia, government, and non-governmental organisations; this enabled triangulation of perspectives which improved the credibility and dependability of the study (38). The rich heterogeneous group enabled in-depth information to be derived thus increasing the trustworthiness of the study findings. Moreover, a rich description of the study participants from the six regions of Somaliland and from the different institutions enables transferability of the study findings to similar settings. To ensure confirmability of the study findings, authors were conscious of likely personal bias and perception throughout the research process and thus practiced reflexivity to ensure neutrality. However, since this is a qualitative study, findings might not be generalizable.

## 6. Conclusion

This study highlights the importance of health research systems in Somaliland. Lack of a national health research institute, policies and priorities, limited funding allocated for health research, inadequate staff with the skills set to conduct research and weak governance systems were identified as barriers to a quality health research system. We recommend the establishment of a national health research institute, development of a national health research policy and priorities, allocation of sufficient and sustainable funding, capacity building of research staff and strengthening of the national health research governance in health research systems. There is a need for multisectoral collaboration among all the stakeholders including government and non-governmental organisations in the establishment and development of the national health research institute and policy to avoid duplication of efforts and promote ownership. Further research is recommended to explore national institutional processes and frameworks for establishing a national health research to enhance health research institutes in Somaliland.

## Data Availability

The original contributions presented in the study are included in the article/Supplementary Material, further inquiries can be directed to the corresponding author.
